# Longitudinal bidirectional association of biological aging acceleration with depressive symptoms in mid-to-late life: evidence from the China Health and Retirement Longitudinal Study

**DOI:** 10.1007/s11357-025-01845-w

**Published:** 2025-08-30

**Authors:** Zeshan Chen, Mengxue Su, Qiang Tu, Jianji Li, Haisheng Wu

**Affiliations:** 1 Department of Burn and Plastic Surgery, People’s Hospital of Shantou, Shantou, Guangdong China; 2 Affiliated Hospital of Youjiang Medical University for Nationalities, Baise, Guangxi China; 3 Key Laboratory of Research on Clinical Molecular Diagnosis for High Incidence Diseases in Western Guangxi of Guangxi Higher Education Institutions, Baise, Guangxi China; 4 Faculty of Medicine and Health, The University of Sydney, Sydney, NSW Australia; 5 School of Public Health, LKS Faculty of Medicine, The University of Hong Kong, 7 Sassoon Road, Hong Kong SAR, China

**Keywords:** Depression, Mental health, Biological aging, Bidirectional association, Cross-lagged panel model

## Abstract

The longitudinal directionality between depressive symptoms and biological aging acceleration has yet to be thoroughly investigated. This study included 5442 Chinese adults aged 45–80 years from the 2011 and 2015 survey waves of China Health and Retirement Longitudinal Study. Multiple biomarker-based biological age was estimated using the Klemera and Doubal method, and biologically older was defined as biological age larger than chronological age. Depressive symptoms were identified using a threshold of ≥ 10 on the 10-item Center for Epidemiological Studies Depression Scale. Multivariate logistic regression was employed to explore two unidirectional associations between biological aging and depression. Cross-lagged panel models (CLPM) were also constructed to simultaneously examine the bidirectional relationship and the strength of the association. In the logistic regression model adjusted for potential confounders, biologically older at baseline was associated with a higher risk of subsequent depression (OR = 1.202, 95% CI: 1.020, 1.417) compared with biologically younger; conversely, individuals with baseline depression had a higher risk of being biologically older later (OR = 1.372, 95% CI: 1.148, 1.639) when compared to those without depression. CLPM identified bidirectional relationship over time, with standardized coefficients of 0.03 (*P* < 0.01) for both longitudinal directional pathways, suggesting an equal contribution of biological aging acceleration and depression to their dynamic interplay. This study reveals a reciprocal interaction between biological aging acceleration and depression in mid-to-late life, suggesting that targeted interventions aimed at decelerating biological aging or alleviating depressive symptoms may confer reciprocal benefits over time.

**Graphical Abstract:**

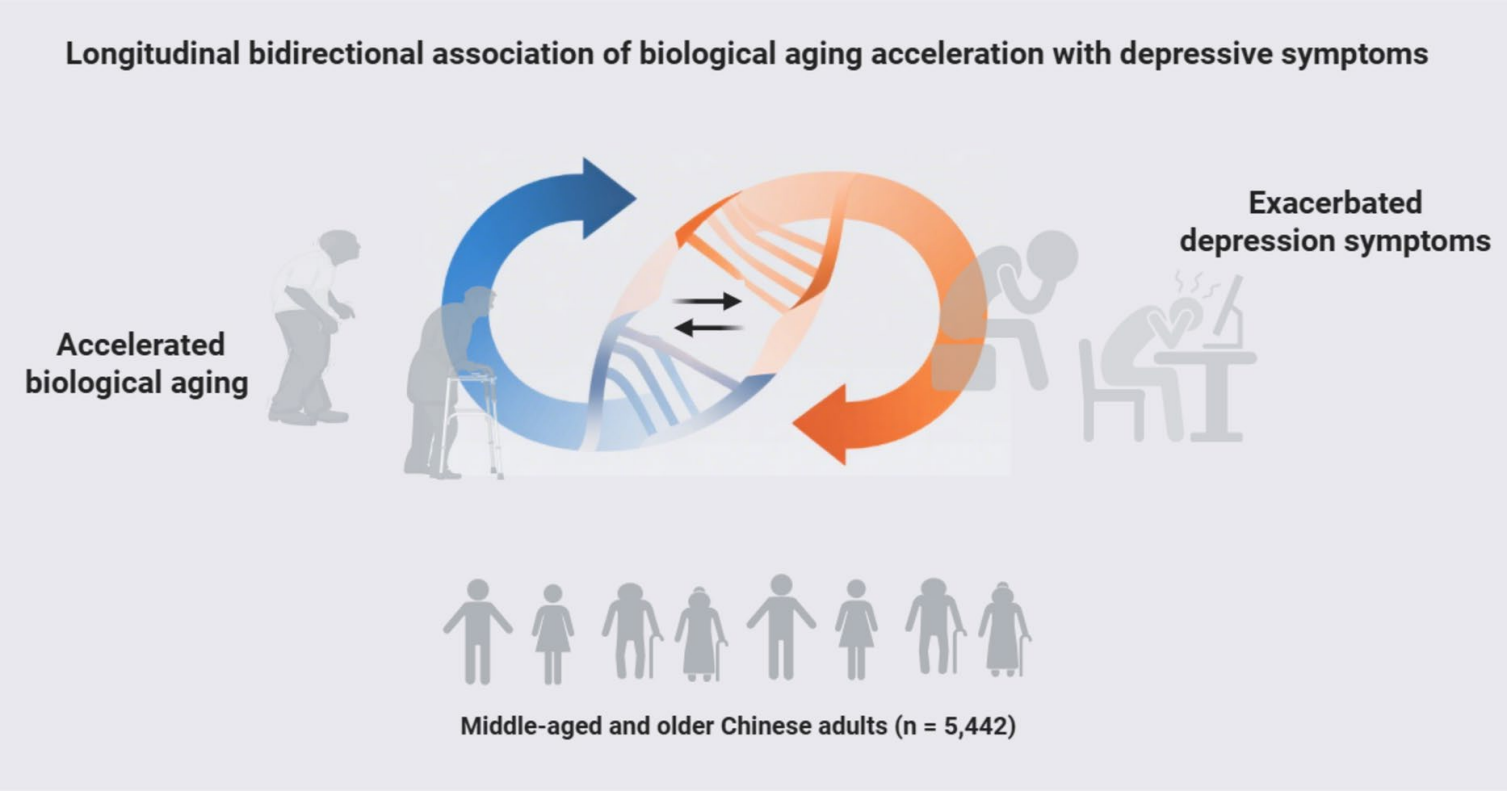

**Supplementary Information:**

The online version contains supplementary material available at 10.1007/s11357-025-01845-w.

## Introduction

Currently, China is facing the challenge of rapid population aging; the elderly population is projected to rise to 400 million by 2050 [1]. Under the context of population aging, emerging studies are increasingly focusing on the concept of biological aging (BA). BA stems from the accumulated changes at the hierarchically organized levels in the human body and is characterized by the progressive decline in physiological functions [2]. Therefore, individuals with the same chronological age may exhibit different levels of acceleration or attenuation in BA, displayed by varying aging-related symptoms and diseases [3]. Recent studies proposed multi-biomarker algorithms of BA that aggregate indices of different biological functions, which could perform well in capturing essential characteristics in the aging process and predicting adverse health outcomes [4–6].

Depression is a prevalent mental disorder that is related to elevated disability and mortality, especially among the elderly [7]. Globally, over 300 million people are affected by depression, with prevalence rates peaking in middle-aged and older adults aged 55–74 years (exceeding 7.5% in females and 5.5% in males) [7]. In China, the surge in mid-to-late life depression prevalence poses a looming threat to its health system [8]. Several cohort studies reported depressive symptoms as a risk factor for faster BA, including self-reported unsuccessful aging [9], brain aging acceleration [10], shorter telomere length [11], epigenetic aging acceleration [12], and faster pace of BA measured by clinical biomarkers [13]. Instead, the reverse pathways from BA to depressive symptoms may also occur but remain incompletely explored. Theory suggests that BA processes may contribute to worsening mental health, including depression, which may be driven by age-related pathophysiological changes [14]. Additionally, the co-occurrence of BA and depression among middle-aged and older people indicates the existence of some potential common biological and behavioral pathways, such as environmental pollution exposure [15–18], intestinal flora [19], white matter hyperintensities, and small cerebral infarcts [20, 21]. However, a few studies have merely assessed the unidirectional association of depression with BA, while there is still an evidence gap of real bidirectionality of the association from the same longitudinal cohort study that can measure both BA and depression symptoms over time. Furthermore, the potential role of demographic and lifestyle factors in modifying the different directional associations remains unexplored.

To address the knowledge gap, this study examined data from the China Health and Retirement Longitudinal Study (CHARLS), a nationally representative, longitudinal cohort, to clarify the temporal bidirectional association between depression and biological aging in Chinese middle-aged and older adults. Additionally, this study also examined vulnerable populations and modifiable lifestyle risk factors for each potential directional association.

## Methods

### Study population

The study was based on CHARLS, details of which have been described in previous publications [22]. Briefly, CHARLS is a comprehensive, ongoing nationwide longitudinal survey targeting middle-aged and elderly Chinese adults aged 45 and older, covering 150 counties/districts in 28 provinces. CHARLS recruited 17708 participants at the baseline surveys in 2011 and has since completed five survey waves. CHARLS has obtained ethical approval from the Biomedical Ethics Review Committee of Peking University (IRB00001052-11015), and all participants were provided with informed written consent.

**Fig. 1:**
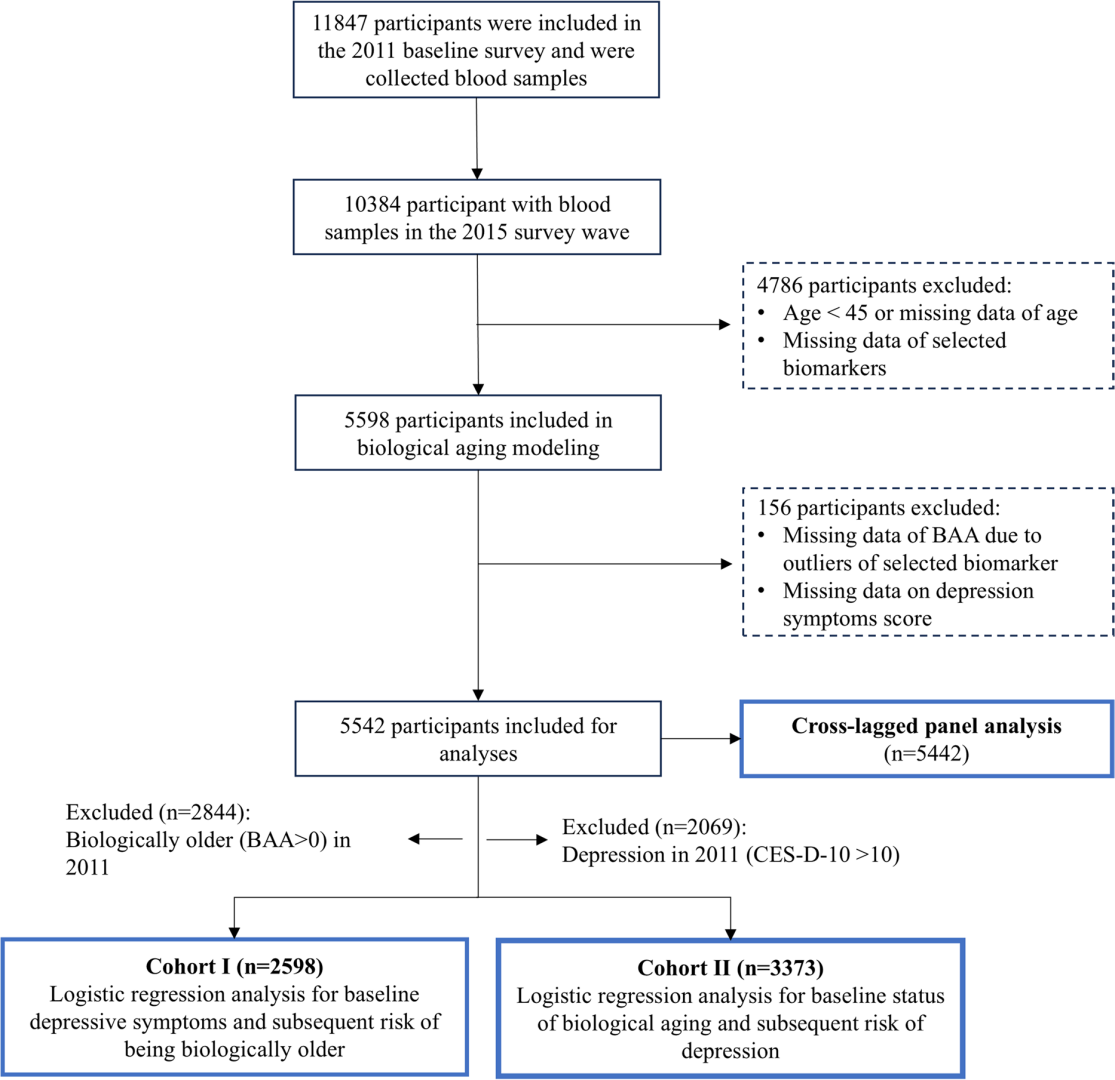
The workflow of this study. Abbreviations: BAA, biological age acceleration based on Klemera and Doubal method (KDM), calculated as KDM biological age minus chronological age; CES-D-10, scores on the 10-item Centre for Epidemiological Studies Depression Scale

In this study, we initially screened 10384 participants with blood biospecimens across both the 2011 and 2015 survey waves. Then, 4786 participants were excluded due to missing data for selected biochemical biomarkers, or because they were younger than 45 or older than 80 years. The exclusion criteria were designed to focus on an age range (45–80 years) where discernible age-related clinical biomarker alterations are typically observed [6, 15]. Subsequently, outliers falling outside the range of mean ± 3 standard deviations (SD) of each selected biomarker were excluded from estimating KDM-biological age [16], leading to 4 participants lacking KDM biological age. Furthermore, 152 participants without depressive symptoms assessments were excluded, resulting in a final sample of 5442 participants for the analyses. Figure 1 displays the exclusion process of study subjects.

### Assessment of depressive symptoms

The depression symptoms were assessed via the 10-item Center for Epidemiological Studies Depression Scale (CES-D-10), which has been recognized for its excellent performance in identifying people with depressive symptoms [23]. Notably, CES-D-10 has also been reported to have great sensitivity in Chinese older adults [24]. The CES-D-10 includes 10 items, each with four response options scored from 0 to 3; the total CES-D-10 score ranges from 0 to 30, with higher scores indicating more severe depressive symptoms. According to previous studies, the cutoff point of 10 was used for identifying depression among participants in CHARLS [17, 25–27]. Therefore, we divided participants into a depression group and a non-depression group based on whether their CES-D-10 score was greater than 10.

### Covariates

In this study, covariates were categorized into constant and time-varying variables. The constant covariates included sex (male or female), education level (no formal education, less than high school, or high school and above), and residence (rural or urban). The time-varying covariates included age (years), body mass index (BMI, kg/m^2^), marital status (married or other [partnered, separated, divorced, widowed, or never married]), alcohol consumption (never, occasional [≤ 3 times/week], or regular [> 3 times/week]], smoking status (never or ever), engagement in social activities (yes or no), household cooking fuels (clean [gas, liquefied petroleum gas, biogas, electricity, or solar energy], or solid [wood, coal, or crop residues]), and personal earnings after tax (positive or non-positive).

### Assessment of biological aging

Ideally, a measurement of biological age should capture the aging processes across multiple biological systems [28, 29]. The Klemera and Doubal method (KDM), integrating information from a set of clinical biomarkers, has proven to be one of the most effective indices of biological age [30]. KDM biological age has also been validated across multi-ethnic cohorts [31] and within the Chinese population [5, 6, 15, 16, 18, 32] for its good performance in capturing age-related health outcomes. The criteria and principles for selecting biomarkers, calculation formulas, and coding for the KDM biological age algorithm have been detailed in published literature [33] and the corresponding R package (https://github.com/dayoonkwon/BioAge).

Notably, there is no unified consensus regarding the selection of biomarkers for constructing the KDM algorithm across previous studies, which have commonly chosen appropriate and available biomarkers tailored to specific study contexts [5, 6, 18, 31, 34]. Following recent CHARLS-based studies [15, 18], we selected 11 clinical biomarkers to calculate KDM biological age: high-sensitivity C-reactive protein (hs-CRP), white blood cell count (WBC), platelet count (PLT), peak expiratory flow (PEF), triglycerides (TG), total cholesterol (TC), glycated hemoglobin, creatinine, blood urea nitrogen (BUN), mean corpuscular volume, and systolic blood pressure (SBP). These biomarkers reflect a range of physiological functions, including inflammatory, immune, metabolic, cardiovascular, pulmonary, hepatic, renal, and hematologic systems. TG and hs-CRP, which exhibited non-normal distributions, were log-transformed before fitting the KDM model.

Based on the "BioAge" R package version 0.1.0 [33], the KDM algorithm was fitted separately by sex. The KDM biological age was calculated using a series of regression analyses that correlate individual biomarkers with chronological age; then, we further calculated biological age acceleration (BAA), defined as KDM biological age minus chronological age, to reflect the variations in the biological aging process among participants with the same chronological age [18, 31]. A BAA greater than 0 suggests a participant was biologically older than what is typical for their chronological age, while a BAA less than 0 suggests they were biologically younger than expected.

### Validation analyses of biological aging indicators

Given that all study subjects participated in both 2011 and 2015 survey waves and that the effectiveness of biological age indicators is usually evaluated by their ability to predict future mortality risk [6, 31, 35], we first trained the KDM algorithm using the data of 2015 survey wave. Then, the fitted KDM model was applied to the data in the 2011 survey wave. The validation analyses for biological aging indices included two parts: (1) examining the Pearson correlation coefficient (*R*) between KDM biological age and chronological age; (2) examining the association of BAA at the 2015 survey wave with subsequent risk of all-cause mortality between 2015 and 2020 by hazard ratio (HR) from Cox proportional hazard models. Consistent with previous studies [6], two Cox models with different adjustment levels were fitted: (1) model 1 was adjusted for age and sex; (2) model 2 was additionally adjusted for BMI, education, marital status, residence, alcohol consumption, and smoking history.

### Statistical analyses

We presented baseline characteristics of participants categorized by biological aging status (biologically older vs. biologically younger) and depression groups (yes or no) at baseline. Continuous variables (age, KDM biological age, BMI) were expressed as means with standard deviations (SD), with group comparisons made using the *t* test. Categorical variables were presented as counts and percentages, with group comparisons conducted using the chi-square test.

#### Longitudinal unidirectional analyses

Considering this study included the 2011 survey wave and 2015 survey wave with a fixed follow-up duration of 4 years, logistic regression models were used to explore the relationship between baseline biological aging and follow-up depression status among the participants without depression at baseline (CES-D-10 ≥ 10) (*n* = 3373). Similarly, logistic regression models were also performed to examine the association of baseline depressive symptoms with the subsequent risk of biological aging acceleration among those participants who were not identified as biologically older (BAA > 0) at baseline (*n* = 2598). Four models with sequential adjustments were established: model 1 was the unadjusted model; model 2 was adjusted for basic demographics including age, sex, BMI, residence, education level, and marital status; model 3 was adjusted for model 2’s covariates and for behavioral and lifestyle factors, including alcohol consumption, smoking status and engagement in social activities; model 4 (fully adjusted model) was adjusted for model 3’s covariates and for proxy variables of income levels, including household cooking fuels, and personal earnings after tax. Multiple imputation by chained equations (MICE) was employed to handle missing covariate data.

Additionally, we also performed stratification analysis to explore whether each of the two unidirectional relationships was modified by demographic characteristics and lifestyle factors, including age, sex, BMI, educational level, marital status, residence, alcohol consumption, smoking history, and social activity engagement. Effect modification was assessed with two-sample *z* test, by examining the difference in effect estimates across stratification along with their 95% CIs [36]; the formula used was $({Q}_{1}-{Q}_{2})\pm 1.96\sqrt{{se}_{1}^{2}+{se}_{2}^{2}}$, where ${Q}_{1}$ and ${Q}_{2}$ represent the regression coefficients for the respective stratification, and ${se}_{1}$ and ${se}_{2}$ are their standard errors. The *P* value from *z* tests was applied to determine the significance of effect modification.

#### Cross-lagged panel analysis

To investigate the bidirectional relationship between biological aging acceleration and depressive symptoms simultaneously, we constructed a cross-lagged panel model (CLPM) among the total participants (*n* = 5442). This modeling framework explored the reciprocal association of the two factors over time after accounting for time-invariant and time-varying confounding [37, 38]. Moreover, the CLPM enables comparison of directional association strengths via standardized weighted coefficients, identifying the predominant drivers of this bidirectional interplay. We constructed a fully adjusted CLPM, adjusting for all baseline covariates (age, sex, BMI, residence, education level, marital status, alcohol consumption, smoking status, engagement in social activities, household cooking fuel, and personal earnings after tax) and corresponding time-varying covariates at the follow-up (age, BMI, marital status, alcohol consumption, smoking status, engagement in social activities, household cooking fuel, and personal earnings after tax). The CLPM estimated standardized coefficients reflecting the strength and direction of relationships over time, including both autoregressive (within-variable) and cross-lagged (between-variable) coefficients. These coefficients quantify how changes in one variable at a given time point predict subsequent changes in the same or another variable, adjusting for potential confounders and concurrent correlations between variables [26]. Full information maximum likelihood estimation with robust (Huber–White) standard errors was applied to address the incomplete data on covariates [39].

### Sensitivity analyses

Sensitivity analyses were conducted to examine the robustness of the results. First, we repeated the CLPM using the multiple imputations by chained equations (MICE) to impute the missing covariate data. Then, to be consistent with the longitudinal unidirectional analysis, we repeated the CLPM analysis by only controlling the baseline covariates.

All statistical analyses were performed using R software (version 4.3.1). Two-sided tests with *α* = 0.05 were considered statistically significant.

## Results

### Participant characteristics

Table 1 presents the baseline characteristics of the total cohort of 5442 participants, with a mean age of 58.26 ± 7.73 years. Of the participants, 2937 (54.0%) were female, 1471 (27.0%) were illiterate, and 3662 (67.3%) were rural residents. At baseline, 52.3% of participants were identified as biologically older, while 38.0% reported depressive symptoms. Compared to those identified as biologically younger, biologically older participants were more likely to be male, have lower levels of education, and have a history of smoking (all *P* values < 0.05). The baseline depression prevalence was higher among females, rural residents, and those who were unmarried, had low levels of education, did not consume alcohol, had no smoking history, lacked social engagement, used solid cooking fuels, and had no personal earnings after taxes (all *P* values < 0.05).

**Table 1:** Baseline characteristics of the study subjects

Variables	Overall	Biological age			Depression		
		Biologically younger	Biologically older	*P* value	No	Yes	*P* value
No., (%)	5442	2598 (47.7)	2844 (52.3)		3373 (62.0)	2069 (38.0)	
Age, mean ± SD, year	58.2 ± 7.73	58.73 ± 7.70	57.83 ± 7.74	< 0.001	57.91 ± 7.77	58.84 ± 7.64	< 0.001
KDM biological age, mean ± SD, year	58.39 ± 7.91	56.99 ± 7.76	59.68 ± 7.83	< 0.001	57.94 ± 7.94	59.14 ± 7.81	< 0.001
BMI, mean ± SD, kg/m^2^	23.77 ± 3.70	23.70 ± 3.61	23.83 ± 3.79	0.208	23.98 ± 3.63	23.43 ± 3.79	< 0.001
Sex, *n* (%)				0.032			< 0.001
Male	2505 (46.0)	1156 (44.5)	1349 (47.4)		1748 (51.8)	757 (36.6)	
Female	2937 (54.0)	1442 (55.5)	1495 (52.6)		1625 (48.2)	1312 (63.4)	
Education, *n* (%)				< 0.001			< 0.001
No formal education	1471 (27.0)	672 (25.9)	799 (28.1)		767 (22.7)	704 (34.0)	
Less than high school	3467 (63.7)	1646 (63.4)	1821 (64.0)		2217 (65.7)	1250 (60.4)	
High school and above	504 (9.3)	280 (10.8)	224 (7.9)		389 (11.5)	115 (5.6)	
Household registration, *n* (%)				0.115			< 0.001
Urban	1780 (32.7)	822 (31.6)	958 (33.7)		1208 (35.8)	572 (27.6)	
Rural	3662 (67.3)	1776 (68.4)	1886 (66.3)		2165 (64.2)	1497 (72.4)	
Marital status, *n* (%)				0.846			< 0.001
Other	727 (13.4)	350 (13.5)	377 (13.3)		367 (10.9)	360 (17.4)	
Married	4715 (86.6)	2248 (86.5)	2467 (86.7)		3006 (89.1)	1709 (82.6)	
Alcohol consumption, *n* (%)				0.228			< 0.001
Never	3620 (66.5)	1728 (66.5)	1892 (66.5)		2136 (63.3)	1484 (71.7)	
Occasional (< 3 times/week)	896 (16.5)	427 (16.4)	469 (16.5)		595 (17.6)	301 (14.5)	
Regular (> 3 times/week)	651 (12.0)	297 (11.4)	354 (12.4)		460 (13.6)	191 (9.2)	
Missing	275 (5.1)	146 (5.6)	129 (4.5)		182 (5.4)	93 (4.5)	
Smoking status, *n* (%)				0.013			< 0.001
Never	3334 (61.3)	1641 (63.2)	1693 (59.5)		1973 (58.5)	1361 (65.8)	
Ever	2107 (38.7)	956 (36.8)	1151 (40.5)		1399 (41.5)	708 (34.2)	
Missing	1 (0.0)	1 (0.0)	0 (0.0)		1 (0.0)	0 (0.0)	
Engagement in social activities, *n* (%)				0.150			< 0.001
No	2852 (52.4)	1331 (51.2)	1521 (53.5)		1662 (49.3)	1190 (57.5)	
Yes	2589 (47.6)	1266 (48.7)	1323 (46.5)		1711 (50.7)	878 (42.4)	
Missing	1 (0.0)	1 (0.0)	0 (0.0)		0 (0.0)	1 (0.0)	
Household cooking fuel, *n* (%)				0.179			< 0.001
Clean	2138 (39.3)	1050 (40.4)	1088 (38.3)		1503 (44.6)	635 (30.7)	
Solid	3296 (60.6)	1543 (59.4)	1753 (61.6)		1865 (55.3)	1431 (69.2)	
Missing	8 (0.1)	5 (0.2)	3 (0.1)		5 (0.1)	3 (0.1)	
Personal earnings after tax, *n* (%)				0.073			< 0.001
Non-positive	4413 (81.1)	2077 (79.9)	2336 (82.1)		2623 (77.8)	1790 (86.5)	
Positive	1028 (18.9)	520 (20.0)	508 (17.9)		749 (22.2)	279 (13.5)	
Missing	1 (0.0)	1 (0.0)	0 (0.0)		1 (0.0)	0 (0.0)	

### Validation of biological aging indicator

**Fig. 2:**
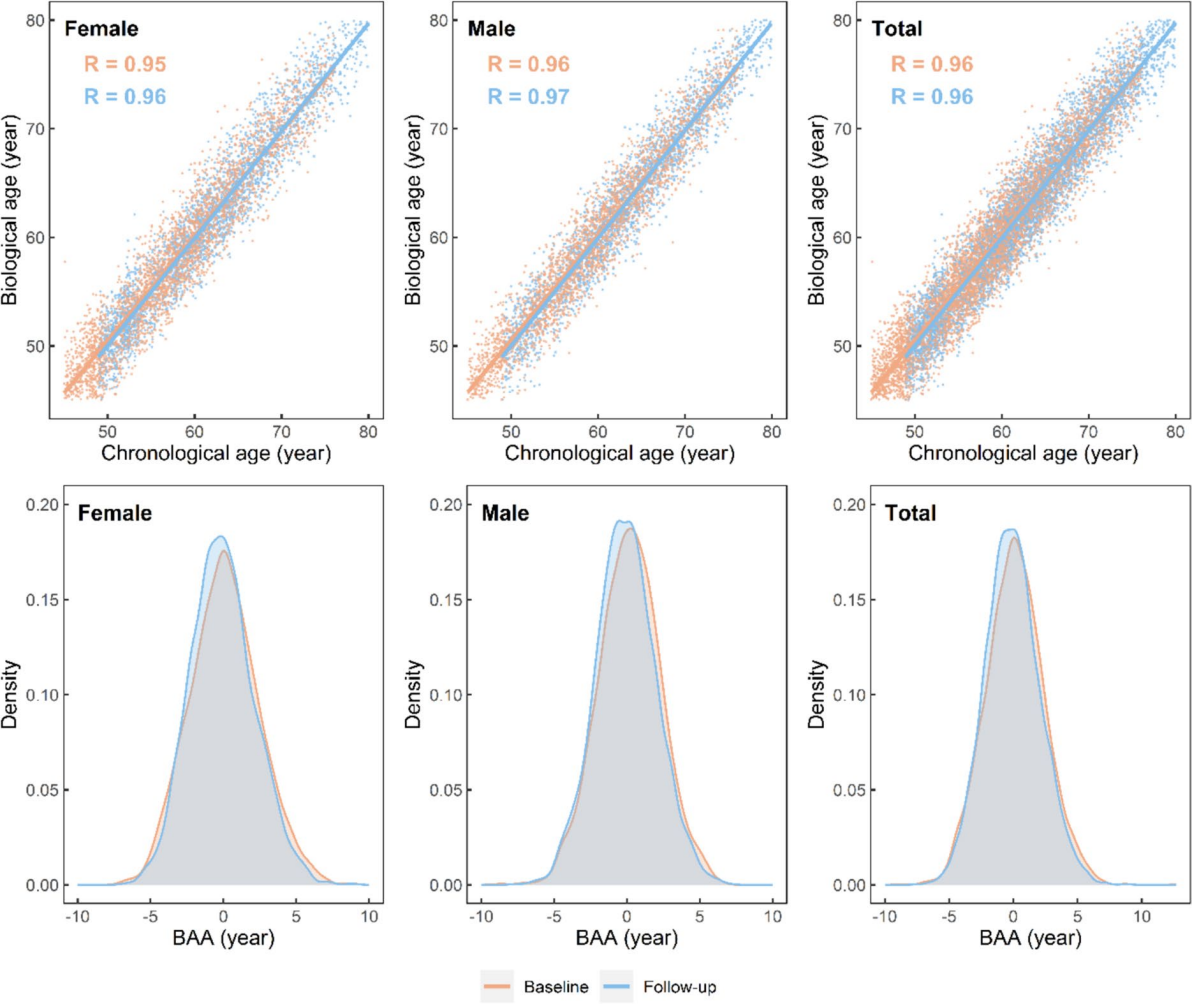
Characteristics of biological age and biological age acceleration (BAA). The top panel displays the scatterplots and fitted lines between biological age and chronological age for female, male, and total participants. The bottom panel shows the distribution of BAA for female, male and total participants. BAA is calculated as biological age minus chronological age. *R* represents the Pearson correlation coefficient

As shown in Fig. 2, the constructed KDM algorithm performed well in fitting chronological age. The correlation coefficient *R* between KDM biological age and chronological age was 0.95 at the 2011 baseline survey and 0.96 at the 2015 follow-up survey among females. For males, KDM biological age was highly correlated with chronological age at baseline (*R* = 0.96) and follow-up (*R* = 0.97). The estimated BAA is mainly distributed within the range of − 5 to 5 years for both males and females.

Table 2 reports the associations of estimated BAA at the 2015 survey wave and all-cause mortality from 2015 to 2020. In the full-adjusted Cox model, per 1-year increase in BAA increased mortality risk by 14% (HR = 1.14, 95% CI: 1.08, 1.20); compared to biologically younger, biologically older was associated with a 56% (HR = 1.56, 95% CI: 1.26, 1.94) increased mortality risk.

**Table 2:** Association of biological aging acceleration in 2015 with all-cause mortality during 2015 and 2020

BAA	Female				Male				Total			
	Participant (*n*)	Death (*n*)	HR (95% CI)	*P*	Participant	Death (*n*)	HR (95% CI)	*P*	Participant	Death (*n*)	HR (95% CI)	*P*
Model 1^a^												
Per 1-year increase in BAA	2937	98	1.13 (1.04, 1.23)	0.006	2505	244	1.16 (1.09, 1.24)	< 0.001	5442	342	1.15 (1.10, 1.21)	< 0.001
Categorical												
Biologically younger (BAA < 0)	1557	39	Ref	–	1301	102	Ref	–	2858	141	Ref	–
Biologically older (BAA > 0)	1380	59	1.76 (1.17, 2.64)	0.006	1204	142	1.57 (1.22, 2.03)	< 0.001	2584	201	1.63 (1.31, 2.02)	< 0.001
Model 2^b^												
Per 1-year increase in BAA	2937	98	1.12 (1.03, 1.22)	0.012	2505	244	1.15 (1.08, 1.22)	< 0.001	5442	342	1.14 (1.08, 1.20)	< 0.001
Categorical												
Biologically younger (BAA < 0)	1557	39	Ref	–	1301	102	Ref	–	2858	141	Ref	–
Biologically older (BAA > 0)	1380	59	1.73 (1.15, 2.60)	0.008	1204	142	1.48 (1.15, 1.92)	0.003	2584	201	1.56 (1.26, 1.94)	< 0.001

*BAA*, biological age acceleration, equating to Klemera and Doubal method-based biological age minus chronological age; *HR*, hazard ratio; *CI*: confidence interval

^a^Cox model 1 was adjusted for age, and sex

^b^Cox model 2 was additionally adjusted for BMI, education, marital status, residence, alcohol consumption, and smoking history

### Dynamics of depressive symptoms and biological aging acceleration

**Fig. 3:**
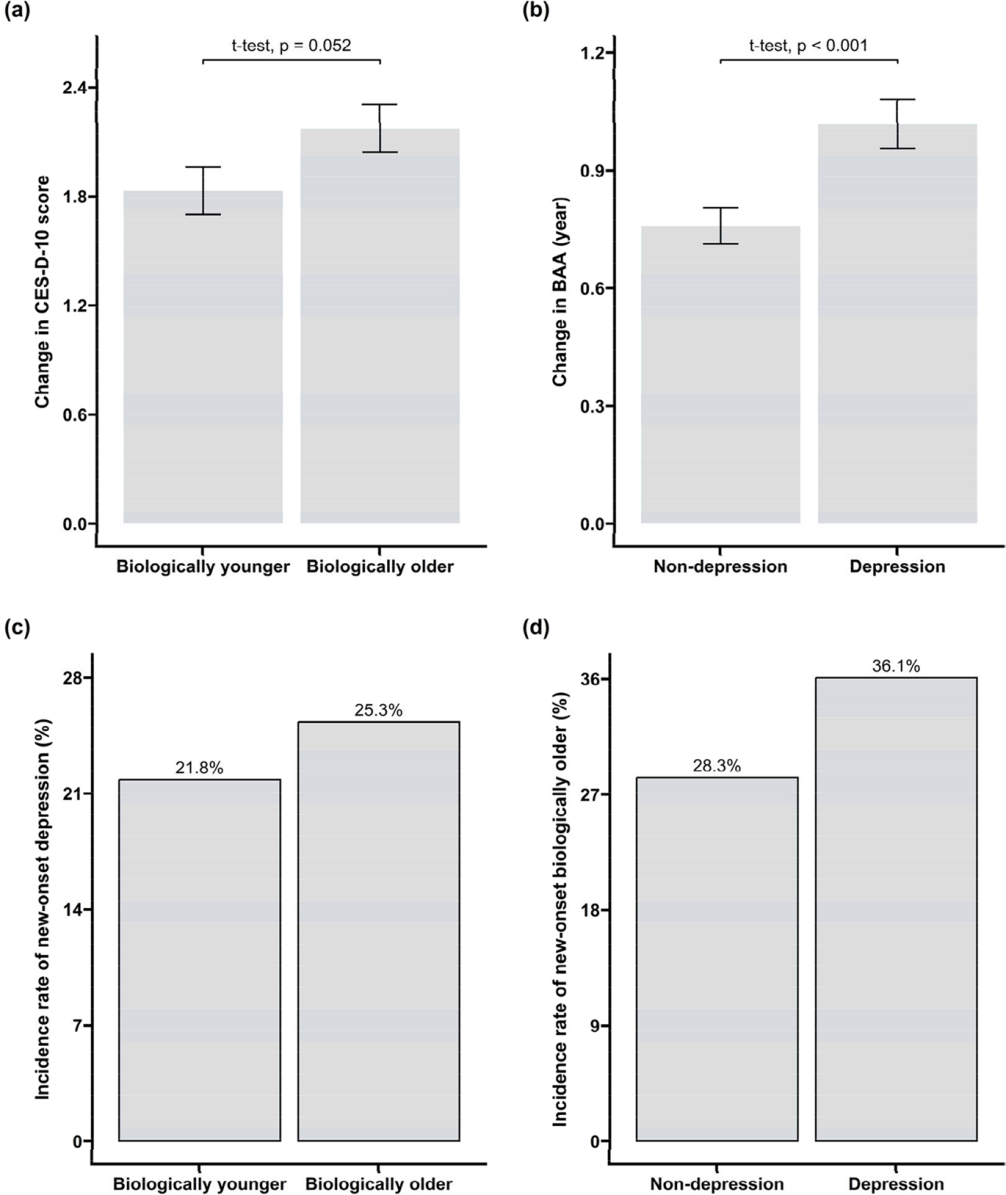
Dynamics of depressive symptoms and biological aging acceleration across different baseline groups, from 2011 to 2015. **a** The change in CES-D-10 score among participants without baseline depression (CES-D-10 ≥ 10), stratified by baseline biological aging status. The change in CES-D-10 was calculated by subtracting the CES-D-10 score in 2015 from the CES-D-10 score in 2011. **b** The changes in biological age acceleration (BAA) among participants who were not biologically older (BAA > 0) at baseline, stratified by baseline depression group. BAA is calculated as biological age minus chronological age. The change in BAA was calculated by subtracting the BAA in 2015 from the BAA in 2011. **c** The incidence rates of new-onset depression by baseline biological aging status. **d** The incidence rates of new-onset biologically order by baseline depression status

Figure 3 illustrates the dynamics of depressive symptoms and biological aging acceleration across different baseline groups during the study period. Among participants without baseline depression, being biologically older at baseline was related to a higher subsequent increase in CES-D-10 score compared to those who were biologically younger (*P* = 0.052), as well as a higher likelihood of developing incident depression (incident rate: 25.3% vs. 21.8%, *P* < 0.001). Similarly, among participants who were not identified as biologically older at baseline, baseline depression significantly led to a higher subsequent increase in BAA (*P* < 0.001) and a higher likelihood of being biologically older later (incident rate: 36.1% vs. 28.3%, *P* < 0.001) compared to those without baseline depression.

### Influence of baseline biological aging acceleration on follow-up depressive symptoms

As shown in Table 3, the logistic regression analysis of 3373 participants without baseline depression revealed that initial biological aging acceleration was significantly associated with an increased risk of subsequent depression. In the fully adjusted model (model 4), participants classified as biologically older at baseline, compared to those classified as biologically younger, had a 20.2% increased risk (OR = 1.202, 95% CI: 1.020, 1.417) of developing depression.

**Table 3:** Unidirectional association between biological aging acceleration and depressive symptoms

Model	Effect of being biologically older at baseline on follow-up risk of depression (*n* = 3373)		Effect of baseline depression status on follow-up risk of being biologically older (*n* = 2598)	
	OR (95% CI)	*P* value	OR (95% CI)	*P* value
Model 1^a^	1.214 (1.035, 1.424)	0.017	1.430 (1.205, 1.696)	< 0.001
Model 2^b^	1.225 (1.040, 1.443)	0.015	1.389 (1.164, 1.657)	< 0.001
Model 3^c^	1.217 (1.033, 1.434)	0.018	1.373 (1.150, 1.640)	< 0.001
Model 4^d^	1.202 (1.020, 1.417)	0.022	1.372 (1.148, 1.639)	< 0.001

^a^Model 1 is the unadjusted model

^b^Model 2 is adjusted basic demographics including age, sex, BMI, residence, education level, and marital status

^c^Model 3 is adjusted for model 2’s covariates and for behavioral and lifestyle factors, including alcohol consumption, smoking status, and engagement in social activities

^c^Model 4 (fully adjusted model) is adjusted for model 3’s covariates and for proxy variables of income levels, including household cooking fuel, and personal earnings after tax

*OR*, Odds ratio; *CI* confidence interval

In the stratification analyses, a marginally statistically significant modification effect for sex was identified in the longitudinal association between baseline biological aging acceleration and later depression. The ORs were 1.03 (95% CI: 0.80, 1.32) for males and 1.36 (95% CI: 1.10, 1.69) for females (*P* for effect modification = 0.095). Notably, a significant modification effect of education level was observed. The estimated ORs were more pronounced among participants with no formal education (OR = 1.54, 95% CI: 1.12, 2.12) compared to those with less than a high school education (OR = 1.06, 95% CI: 0.86, 1.30), with *P* for effect modification = 0.049. No modification effects were found among other factors (Table 4).

**Table 4:** Stratification analysis for the directional associations between depressive symptoms and biologically aging acceleration

	Biologically older → depression		Depression → biologically older	
	OR (95% CI)	*P* for effect modification^a^	OR (95% CI)	*P* for effect modification^a^
Age (years)				
45–59	1.25 (1.01, 1.53)	Reference	1.25 (0.98, 1.59)	Reference
≥ 60	1.16 (0.89, 1.52)	0.684	1.55 (1.18, 2.03)	0.246
Sex				
Male	1.03 (0.80, 1.32)	Reference	1.37 (1.03, 1.82)	Reference
Female	1.36 (1.10, 1.69)	0.095	1.39 (1.11, 1.76)	0.929
BMI (kg/m^2^)				
< 24	1.31 (1.05, 1.64)	Reference	1.45 (1.16, 1.83)	Reference
≥ 24	1.09 (0.85, 1.40)	0.283	1.25 (0.93, 1.67)	0.417
Education level				
Illiterate	1.54 (1.12, 2.12)	Reference	1.64 (1.17, 2.29)	Reference
Less than high school	1.06 (0.86, 1.30)	0.049	1.27 (1.02, 1.58)	0.215
High school and above	1.90 (1.02, 3.54)	0.552	1.45 (0.70, 3.01)	0.774
Marital status				
Other	1.10 (0.69, 1.77)	Reference	1.03 (0.64, 1.65)	Reference
Married	1.21 (1.02, 1.45)	0.713	1.44 (1.18, 1.74)	0.198
Residence				
Urban	1.18 (0.87, 1.59)	Reference	1.16 (0.83, 1.63)	Reference
Rural	1.24 (1.01, 1.50)	0.806	1.47 (1.19, 1.81)	0.251
Alcohol consumption				
Never	1.22 (1.00, 1.48)	Reference	1.39 (1.13, 1.71)	Reference
Occasional (< 3 times/week)	1.23 (0.83, 1.82)	0.956	1.75 (1.11, 2.74)	0.369
Regular (> 3 times/week)	1.14 (0.71, 1.85)	0.814	0.85 (0.48, 1.51)	0.114
Smoking history				
Never	1.29 (1.04, 1.58)	Reference	1.50 (1.20, 1.88)	Reference
Ever	1.09 (0.83, 1.42)	0.326	1.19 (0.88, 1.61)	0.221

^a^*P* values from the z-test were used to assess effect modification

*BMI*, Body mass index; *OR*, odds ratio; *CI*, confidence interval

### Influence of baseline depressive symptoms on follow-up biological aging acceleration

As shown in Table 3, the logistic regression analysis of 2598 participants who were not biologically older at baseline showed that baseline depressive symptoms were significantly associated with an increased risk of biologically older at follow-up. In the fully adjusted model, participants with depression at baseline had a 37.2% increased risk (OR = 1.372, 95% CI: 1.148, 1.639) of being biologically older compared to those without baseline depression.

In the stratification analyses for the longitudinal effects of baseline depressive symptoms on subsequent biological aging acceleration, we found no significant modification effects by age, sex, BMI, education, marital status, residence location, alcohol consumption, and smoking (all *P* values for effect modification > 0.05) (Table 4).

### Longitudinal bidirectional association between biological aging acceleration and depression symptoms

**Fig. 4:**
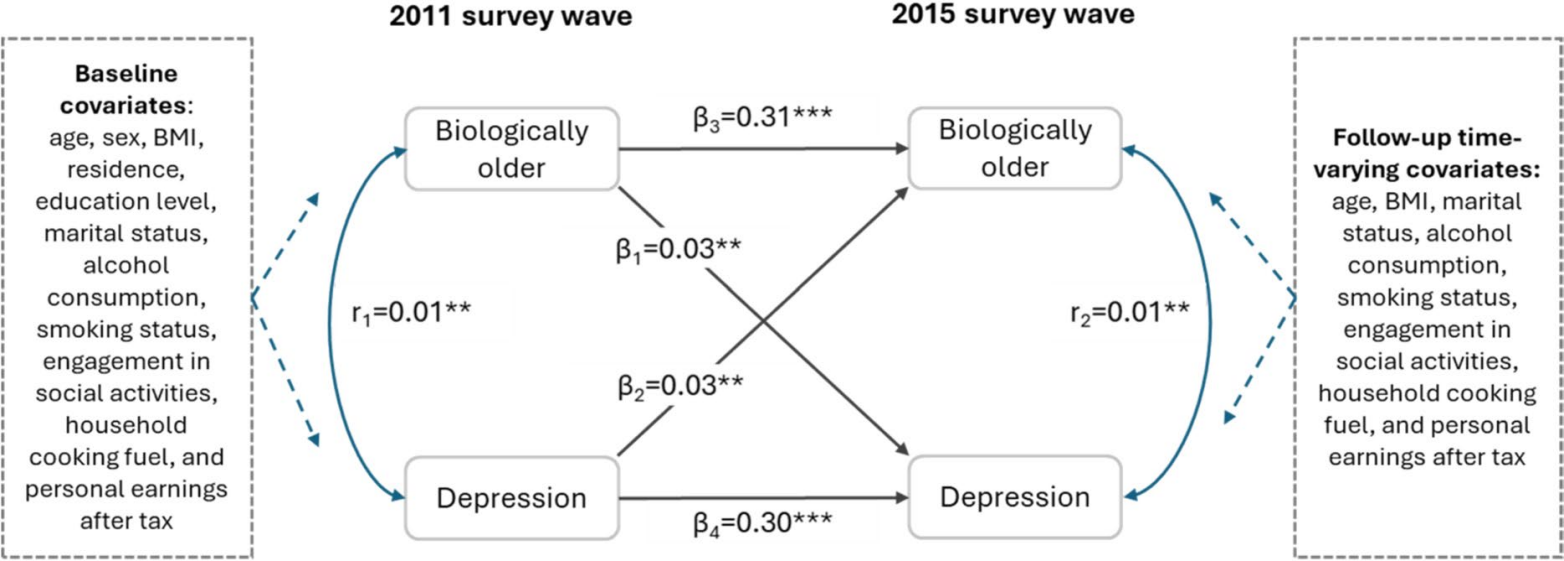
Cross-lagged panel model of biological aging acceleration and depressive symptoms. Biological aging acceleration was treated as binary variables (0 = biologically younger [BAA < 0], 1 = biologically older [BAA > 0]), depressive symptoms was treated as binary variables (0 = non-depression [CES-D-10 < 10], 1 = depression [CES-D-10 ≥ 10]). BAA is calculated as biological age minus chronological age. CES-D-10 is the score on the 10-item Centre for Epidemiological Studies Depression Scale. Standardized coefficients were reported. Single-headed arrows represented regression paths. Double-headed arrows represented correlations. Symbol * indicates 0.01 ≤ *P* < 0.05; Symbol ** indicates 0.001 ≤ *P* < 0.01; Symbol *** indicates *P* < 0.001

A two-wave CLPM was further constructed to examine the bidirectionality. Figure 4 displays standardized coefficients of the CLPM. After accounting for the intrawave concurrent correlations between two variables (*r*_*1*_ and *r*_*2*_), interwave correlations of the same variable (*β*_*3*_ and *β*_*4*_) and confounders, baseline biological aging status remained significantly associated with later depressive symptoms (*β*_*1*_ = 0.03, *P* value < 0.01). Similarly, baseline depressive symptoms were significantly associated with an increased risk of being biologically older (*β*_*2*_ = 0.03, *P* value < 0.01). The equal *β*_*1*_ and *β*_*2*_ values indicated that biological aging acceleration and depression contributed equally to their dynamic bidirectional relationship.

### Sensitivity analyses

Sensitivity analyses of the CLPM were conducted to assess the robustness of the findings regarding bidirectional relationships. When the CLPM was constructed using MICE to impute missing covariate data, the effect estimates remained highly consistent with the main findings (Figure S1). Additionally, when the CLPM was only adjusted for baseline covariates, the standardized coefficients were consistent, further supporting the robustness of the main results (Figure S2).

## Discussion

This is the first study to examine the temporal bidirectional relationship between biological aging acceleration and depression using a large population-based sample. Our findings indicated that baseline biological aging acceleration was associated with an increased risk of subsequent depression, thereby supporting the perspective of biological aging as a risk factor for depression. When considering the perspective of biological aging-as-outcome, baseline depression was found to be associated with an increased risk of being biologically older later. Overall, these findings reinforce the emerging perspective that depressive symptoms partly stem from deleterious age-related alterations across multiple physiological systems and that the worsening of depressive symptoms in turn poses risks to the physiological aging acceleration.

The findings of this study are not only largely consistent with previous studies on unidirectional longitudinal association between biological aging and depression, but also extend temporal reciprocal relationships that have not been explored. On the one hand, consistent with our findings on the directional path from baseline depression to subsequent biological aging acceleration, previous studies indicated that baseline depression is unidirectionally positively linked with follow-up measures of biological aging [9–11, 13]. On the other hand, although incompletely studied, previous longitudinal investigations supported the perspective that biological aging acceleration could be a risk factor for depression. For instance, a recent study revealed that elderly adults from the Health, Aging, and Body Composition Study with older KDM-based biological aging had a higher risk of incident depression identified by CES-D-10 score [14]; such unidirectional observation was additionally confirmed by the clinical diagnosis of depression in a larger population with a broader age range from the UK Biobank [34].

**Fig. 5:**
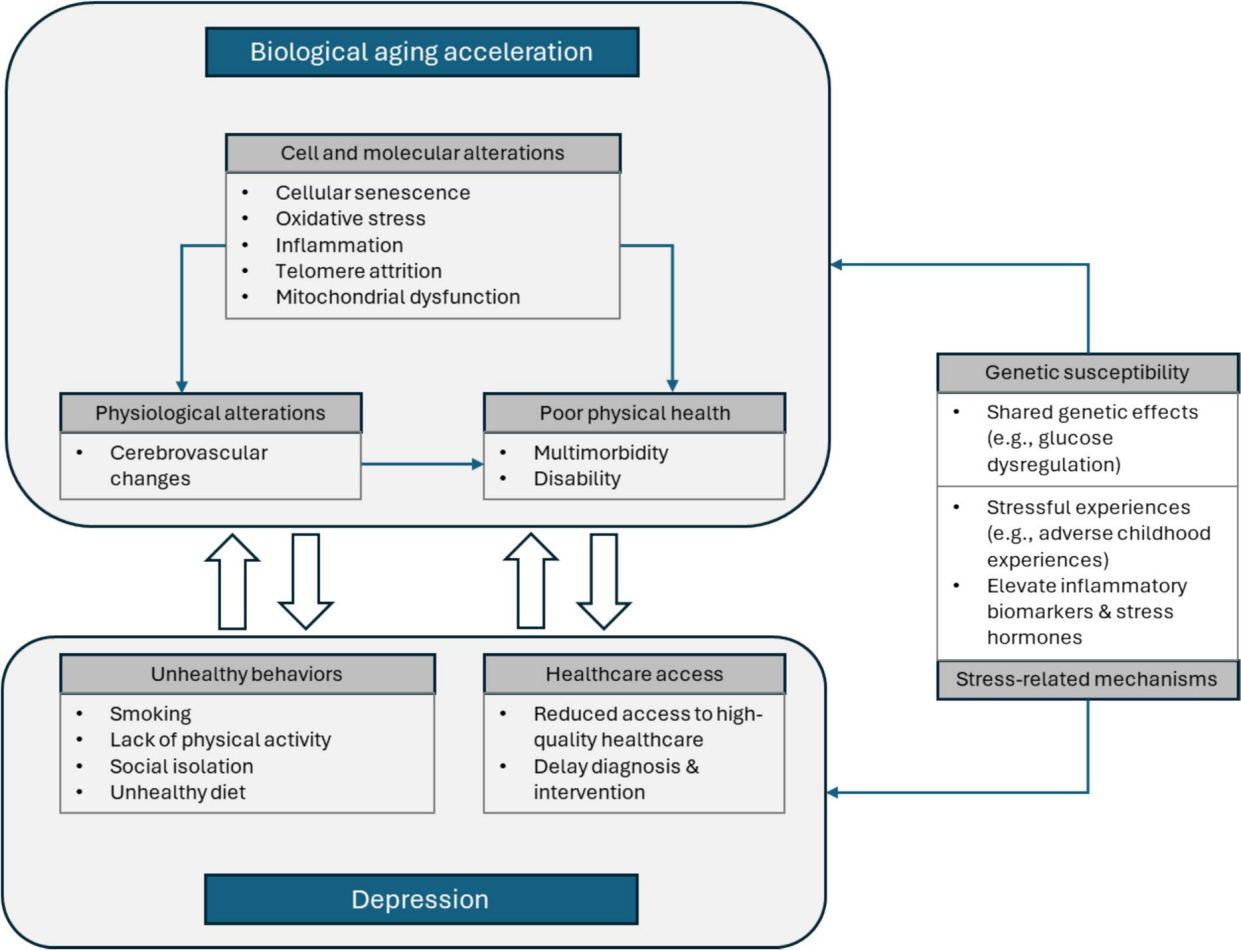
Potential biological mechanisms and behavioral pathways of the bidirectional association between biological aging acceleration and depression

Our findings confirmed the bidirectionality of the association between accelerated biological aging and depression among middle-aged and elderly Chinese adults, but they do not address the mechanisms that may develop at various stages in the life course from the accumulation of molecular alterations to physical impairments and chronic diseases. There are several potential biological mechanisms for the path from biological aging acceleration to depression (Fig. 5). First, cell and molecular alterations at the roots of aging like cellular senescence, oxidative stress, inflammation, telomere attrition, and mitochondrial dysfunction, directly affect psychological processes that contribute to depressive symptoms [40]. Second, physiological alterations stemming from the molecular roots of biological aging, such as cerebrovascular changes, could initiate depressive symptoms [41]. Additionally, downstream in the aging process, poor physical health (e.g., multimorbidity, disability) could directly influence psychological well-being and lead to depression [42, 43].

Meanwhile, some potential mechanisms and behavioral pathways for the path from depression to biological aging acceleration have also been proposed (Fig. 5). First, individuals with mental disorders tend to have poor health behaviors such as smoking, lack of physical activity and social engagement, and unhealthy diet [44], which may accelerate the rate of biological aging. Second, people with mental disorders are less likely to obtain access to high-quality health care. This not only means they miss out on timely professional assessments and appropriate treatment plans but will lead to delayed diagnosis and intervention for any aging-related problems. As a result, the original physical and psychological distress can persist, worsen, and ultimately contribute to an acceleration of the biological aging process [45]. Third, genetic susceptibility may concurrently elevate an individual’s risk for psychopathology and accelerated aging. This could occur through shared genetic effects on depression and aging risk factors, such as glucose dysregulation [46]. Fourth, depression is linked with stressful experiences such as adverse childhood experiences [47]. Stress could elevate the levels of inflammatory biomarkers and stress hormones, which may serve as a bridge connecting depression and aging-related health outcomes [46].

Our findings on biological aging-depression interplay have public health and clinical implications. Currently, with the development of large language models, biological age can be accurately and conveniently estimated using only health examination reports [48]. Integrating biological aging metrics into mid-to-late life mental health screenings could enable early identification of bidirectional risk, guiding targeted interventions. Community programs might combine anti-aging strategies (e.g., lifestyle adjustment, social hobby engagement) with mental health support to address both pathways, with cultural tailoring—such as reducing help-seeking stigma in Chinese contexts.

There are several strengths and novelties of this study. First, this is by far the first large-scale study that contained repeated measurements of depression symptoms and biological aging, illuminating the temporal pattern of reciprocal interaction between them. Such a study design has been rarely performed due to the unavailability of longitudinal data on biochemical biomarkers that are needed to estimate biological aging indicators. Second, we constructed a well-performed and validated biological aging measure, which provides unique insights for healthy aging-oriented interventions. Additionally, our findings were cross-validated by combining two distinct methods, the multivariate logistic regression model and CLPM, and the findings remain robust within these two methods regardless of huge discrepancies in study samples, design, principle, and bias sources.

However, several limitations of this study should be acknowledged. First, due to limitations in data availability, this study was unable to include all clinical biomarkers typically associated with aging [31]. Nevertheless, the multi-biomarker algorithm of biological aging measure of this study aggregated indicators of inflammatory, immune, metabolic, cardiovascular, lung, liver, kidney, and hematologic functioning, and has been validated by its performance in predicting 5-year mortality risk. Second, although longitudinal analysis is less susceptible to unmeasured time-varying confounders, residual confounding was an inevitable limitation due to the inherent nature of observational studies. Third, for repeated assessments of biological aging, we enrolled participants who participated in both the 2011 baseline and 2015 follow-up surveys. The samples we analyzed may exhibit slower biological aging acceleration or fewer depression symptoms at baseline compared to the general middle-aged and older population. This potential selection bias could be due to those with a higher biological aging acceleration or serious depression symptoms having a higher risk of death or loss of follow-up. Nevertheless, such selection biases tended to attenuate the association estimates toward the null, and our findings were therefore conservative. Fourth, although this study has identified the longitudinal bidirectionality of the association between biological aging acceleration and depression, it could not fully establish causality. Therefore, there is a need for randomized controlled trials or causal inference studies to further explore the temporal interplay between accelerated biological aging and depression. Finally, our participants were all Chinese adults in mid-to-late life, limiting the generalization of the results to other age ranges and ethnic groups. Culturally, the Chinese emphasis on collective harmony may suppress depressive symptom reporting or delay help-seeking, diverging from Western contexts with more open mental health discourse and thus affecting result generalizability. From a healthcare perspective, China’s uneven urban–rural distribution of mental health resources and limited psychological services in primary care differ from regions with universal access to mental healthcare. Given that older Chinese adults reside in rural areas, the specific aging-depression interplay pattern in China may not be applicable to population from other countries.

## Conclusion

In conclusion, we found that biological aging acceleration and depression were bidirectionally associated among middle-aged and older adults. Under the circumstances of population aging, this newly revealed bidirectional relationship underscores that interventions aimed at slowing biological aging or improving mental well-being could bring reciprocal benefits over time. In addition, approximately equal standardized cross-lagged coefficients suggest that biological aging acceleration and depressive symptoms equally drive their dynamic interplay. Further research is needed to elucidate the underlying biological and behavioral mechanisms mediating this bidirectional relationship.

## Supplementary Information

Below is the link to the electronic supplementary material.

Supplementary file1 (DOCX 66 KB)

## Data Availability

Data of the CHALRS can be obtained at https://charls.charlsdata.com/pages/data/111/en.html.
