# Supplementary material for: Longitudinal bidirectional association of biological aging acceleration with depressive symptoms in mid-to-late life: evidence from the China Health and Retirement Longitudinal Study
Source: GeroScience. 2025 Aug 30;48(2):3097–113. doi: 10.1007/s11357-025-01845-w (PMC12972361; doi:10.1007/s11357-025-01845-w)

**Supplementary Online Content**

**Figure S1. Cross-lagged panel model estimates for biological aging acceleration and depression, using MICE to impute missing data on covariates.** Standardized coefficients were reported. Single-headed arrows represented regression paths. Double-headed arrows represented correlations. Covariates include age, sex, BMI, residence, education level, marital status, residence, alcohol consumption, smoking status, engagement in social activities, household cooking fuel, and personal earnings after tax. Symbol * indicates 0.01 ≤ *P* < 0.05; Symbol ** indicates 0.001 ≤ *P* < 0.01; Symbol *** indicates *P* < 0.001.


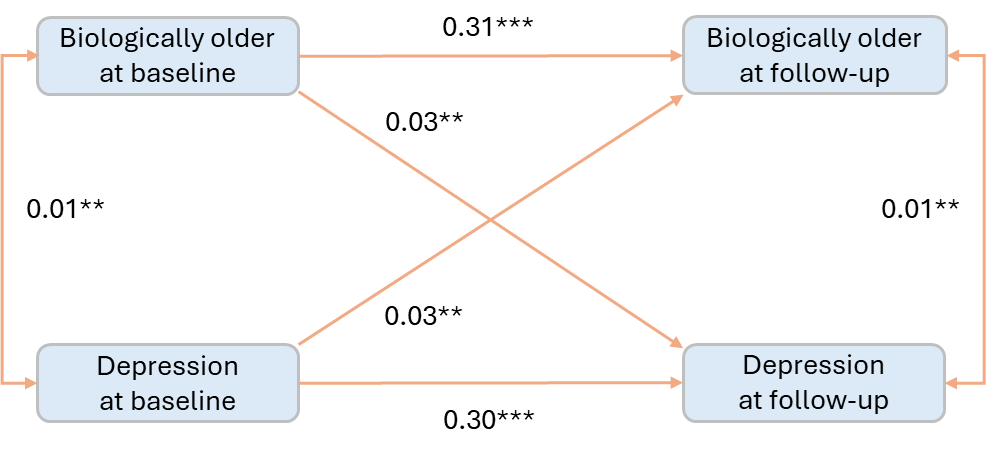


**Figure S2. Cross-lagged model estimates for biological aging acceleration and depression, by adjusting for baseline covariates.** Standardized coefficients were reported. Single-headed arrows represented regression paths. Double-headed arrows represented correlations. Covariates include age, sex, BMI, residence, education level, marital status, residence, alcohol consumption, smoking status, engagement in social activities, household cooking fuel, and personal earnings after tax. Symbol * indicates 0.01 ≤ *P* < 0.05; Symbol ** indicates 0.001 ≤ *P* < 0.01; Symbol *** indicates *P* < 0.001.


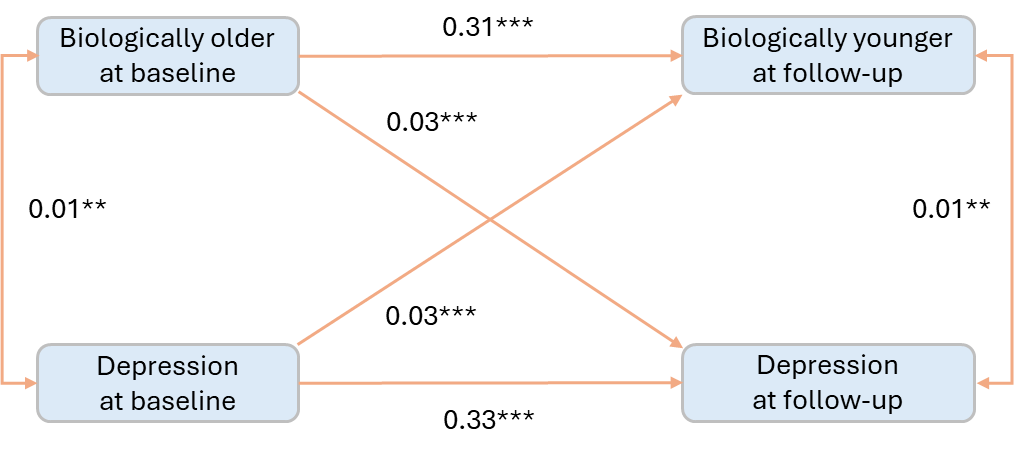

Supplement: Supplementary file 1 — Supplementary file1 (DOCX 66 KB) [file 11357_2025_1845_MOESM1_ESM.docx]
